# 

**Published:** 1896-09-26

**Authors:** 


**CADBURY'S COCOA**
"The standard of highest purity at present attainable."—*Lancet.*

**CADBURY'S COCOA**

NO ALKALIES USED.

### THE HOSPITAL

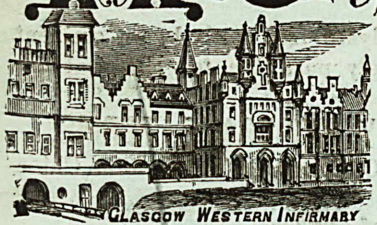

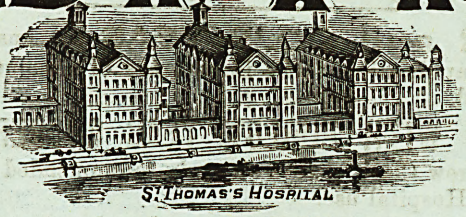

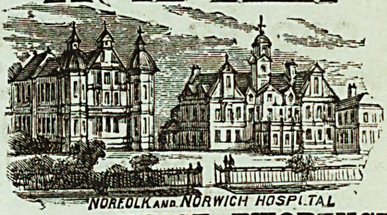

No. 522. Vol. XX.
SATURDAY, SEPT. 26TH, 1896.

WITH EXTRA NURSING SUPPLEMENT.

[Registered at the General Post Office as a Newspaper for transmission in the United Kingdom and Abroad.]

PRICE TWOPENCE.

Per Annum, 10s. 6d., post free.

Monthly Parts, 6d.; post free, 8d.

Ditto per Annum, 8s. 6d., post free.

